# Hepatitis Awareness Month and National Hepatitis Testing Day — May 2013

**Published:** 2013-05-17

In the United States, an estimated 3.5–5.3 million persons have chronic hepatitis B or chronic hepatitis C, and as many as three fourths of those with hepatitis C are unaware they are infected. To increase provider and public awareness of viral hepatitis and the need for testing, May has been designated Hepatitis Awareness Month, and May 19 is recognized as National Hepatitis Testing Day.

Testing of persons to assess current infection with hepatitis C virus, especially those born during 1945–1965 (i.e., “baby boomers”), who have a higher prevalence of chronic hepatitis C than other birth cohorts (1), is an important step in achieving the viral hepatitis prevention goals set forth by the U.S. Department of Health and Human Services (2). CDC also has published updated testing guidance for clinicians and laboratorians to ensure the identification of persons with current hepatitis C virus infection (3).

To promote viral hepatitis awareness beyond Hepatitis Awareness Month, CDC’s Division of Viral Hepatitis will partner with the National Hepatitis B United Coalition (Hep B United) to release a national, multilingual education campaign in June. This campaign will engage community partners to promote hepatitis B virus testing among Asian Americans and other populations experiencing health disparities related to hepatitis B.
